# Authors’ reply to “Left atrial strain: an operator and software-dependent tool”

**DOI:** 10.1186/s13613-024-01332-z

**Published:** 2024-08-17

**Authors:** Marta Cicetti, François Bagate, Cristina Lapenta, Ségolène Gendreau, Paul Masi, Armand Mekontso Dessap

**Affiliations:** 1 AP-HP, Hôpitaux Universitaires Henri Mondor, DHU A-TVB, Service de Médecine Intensive Réanimation, 1 rue Gustave Eiffel, Créteil, F-94010 France; 2 Università Cattolica del Sacro Cuore, Roma, Italy; 3 Faculté de Médecine, Groupe de recherche clinique CARMAS, Université Paris Est Créteil, Créteil, F-94010 France; 4 INSERM U955, Institut Mondor de Recherche Biomédicale, Créteil, F-94010 France

Dear Editor,

We thank Beyls and colleagues for their interest in our recent article on left atrial strain (LAS) [1]. They stated that our sample size calculation, based on a predicted 5% increase in the LAS reservoir (LASr), might be underestimated given that the repeatability and reliability of the LAS measurement is unknown. As stated in the Methods section of our paper, we attempted to minimise measurement inconsistencies. First, we used Automated Function Imaging (AFI) left atrial software (EchoPAC, GE Healthcare), which follows the 2018 EACVI-ASE Strain Standardised Task Force guidelines [2]. Second, we performed LAS analysis according to the cited recommendations [2], including measurement of left atrial strain in both four-chamber and two-chamber apical views. The same methodology has been used in large studies that have helped to define reference values for LAS [3]. Third, we assessed intra-observer variability in 10 randomly selected subjects using the interclass correlation coefficient (ICC), a measure of repeatability according to the study by Bunting et al. [4]. In our cohort, intra-observer analysis showed good repeatability of the three components of left atrial strain: LASr (ICC = 0.92), LAS conduit (LAScd) (ICC = 0.81), LAS contraction (LASct) (ICC = 0.95). Finally, we performed intra- and inter-observer variability analysis according to the methods of previous studies, including the paper by Beyls et al. [5], which investigated LAS as a predictive marker of atrial fibrillation (AF) in patients with COVID-19 pneumonia.

Beyls et al. mentioned that the value of the LASr we identified for sample size calculation was derived from previous studies using different software for LAS analysis and that there is some evidence of inter-vendor variability in strain measurements. They cited the study by Wang et al. [6] comparing EchoPAC version 201 (GE Vingmed Ultrasound) and Image Arena 2D Cardiac Performance Analysis version 4.6 (TomTec Imaging Systems, Unterschleissheim, Germany). However, in the paper by Wang et al. [6], the EchoPAC system used the LV strain package to assess LAS, as did many other studies prior to the commercialisation of the AFI left atrial software. To our knowledge, there are no published studies comparing EchoPAC AFI left atrial and TomTec.

The authors correctly mentioned that assessing fluid responsiveness by Left Ventricular Outflow Tract – Velocity Time Integral (LVOT – VTI) variation may have led to misclassification of patients. We agree that this is a limitation of our work and have already discussed this in the Limitations section of the article. The observational nature of our study necessitated the use of echocardiography to estimate cardiac output, as haemodynamic monitoring is routinely performed by echocardiography in our ICU. Regarding the observed improvement in haemodynamics in the non-responder group, although the increase in arterial pressure during volume expansion was statistically significant, its clinical relevance is questionable.

The authors highlight that we studied a heterogeneous cohort of patients with varying degrees of pre-existing left atrial dysfunction prior to ICU admission; this may have influenced the LAS response to fluid administration. We agree that cardiac comorbidities may affect atrial function in the acute setting and influence treatment response. However, it is difficult to investigate the contribution of pre-admission pathology because baseline echocardiography is not always available and rarely includes left atrial strain measurement. We tested the two groups (fluid responders and non-responders) for differences in comorbidities and there was no significant difference (unpublished). From a pragmatic perspective, as chronic cardiovascular disease is common in critically ill patients, we believe that our cohort reflects the routine practice ICU population.

In conclusion, we agree that further studies are needed to investigate the usefulness of LAS in critically ill patients.

## Data Availability

Not applicable.

## Abbreviations

LAS: Left Atrial Strain
LASr: Left Atrial Strain reservoir
AFI: Automated Function Imaging
EACVI: European Association of CadioVascular Imaging
ASE: American Society of Echocardiography
LAScd: LAS conduit
LASct: LAS contraction
AF: Atrial Fibrillation
LVOT – VTI: Left Ventricular Outflow Tract – Velocity Time Integral
ICU: Intensive Care Unit
